# The relationship between physical activity and depression, anxiety, and stress in college students: a mediating effect of diet

**DOI:** 10.3389/fnut.2025.1611906

**Published:** 2025-10-07

**Authors:** Sheng Jiazhi, Chen Caixia, Gong Lamei, Zhou Jian

**Affiliations:** ^1^Laboratory of Sports and Health Promotion, School of Physical Education, Sichuan University of Arts and Science, Dazhou, China; ^2^School of Physical Education, China West Normal University, Nanchong, China

**Keywords:** physical activity, emotional overeating, satiety, delayed eating, depression-anxiety-stress

## Abstract

**Objective:**

Previous studies have demonstrated that engaging in physical activity is a vital behavioral element in mitigating experiences of depression, anxiety, and stress. However, the specific mechanisms by which dietary practices and physical activity interact to affect these psychological conditions are not fully understood. Thus, this study aimed to comprehensively examine the relationships between physical activity, diet, and mental health outcomes, specifically depression, anxiety, and stress, among college students and to elucidate the pathways through which these factors interact.

**Method:**

This study analyzed 1,076 college students (18.8 ± 0.8 years old) via the Adult Dietary Behavior Questionnaire (covering eight dimensions), the physical activity scale, and the depression-anxiety-stress scale.

**Results:**

There were significant differences in dietary behavior and physical activity parameters between male and female college students; however, no significant differences were observed in levels of depression, anxiety, or stress. The dimensions of college students' dietary behavior were significantly correlated with symptoms of anxiety, stress, and depression (*p* < 0.01). Higher levels of physical activity were negatively correlated with anxiety, stress, and depression symptoms in college-aged students (*p* < 0.01). Dietary behavior was significantly correlated with college students' physical activity. Through structural equation modeling analysis, our findings revealed that physical activity not only directly reduced depression, stress, and anxiety among college students but also indirectly alleviated them by improving their dietary behaviors. The direct effects of physical activity on depression, anxiety, and stress in college students were greater than the mediating effects of dietary behavior.

**Conclusion:**

Regular physical activity plays a key role in reducing depression, anxiety, and stress among college students, with dietary behavior serving as a mediating factor. Therefore, promoting exercise and healthy eating within university settings is recommended for better mental health. Future research should investigate this relationship across diverse populations to gain valuable insights into mental health interventions.

## 1 Introduction

Depression is the second most common disease globally, affecting approximately 3.8% of the population, or 280 million people, with major depression rates varying from 6 to 18% across cultures (1). Anxiety disorders affect approximately 4.4% of the global population, whereas depression and anxiety together represent 38% of the mental illness burden (2, 3). The risk in individuals with diabetes is approximately 78% higher than that in the general population, and anxiety disorder patients have a 65% higher risk of hypertension (4). The mechanism of depression involves genetic, environmental, and biological factors, with genetics contributing to approximately 37% of the risk (5). Psychosocial factors, including childhood trauma and socioeconomic stress, can increase the risk for adult depression (6, 7). Adolescent mental health disorders in China are estimated to have a prevalence of 8.9%, with anxiety (8.2%) and depression (6.1%) accounting for 22.4% of health loss (8).

### 1.1 The role of physical activity in depression, anxiety, and stress

Physical activity is widely recognized for improving health, promoting social interaction, enhancing self-efficacy, benefiting mental health, and alleviating depression (9). Physical activity is considered to be an effective method for the treatment of depression, mainly through the regulation of serotonin. Aerobic exercise can promote the release of 5-hydroxytryptamine in the anterior cingulate cortex to relieve depression, pain, and anxiety. These findings provide a biological basis for the application of physical activity in mental health interventions (10). Physical activity influences serotonin release and receptor function, with exercise enhancing 5-HT1A and 5-HT7 receptor activity linked to reduced depressive symptoms (11). Exercise reduces inflammation, regulates the endocrine system, increases serotonin levels, improves mood, and alleviates depression through increased brain-derived neurotrophic factor and neural plasticity (12). Exercise affects depression by regulating neurotransmitters, reducing inflammation, and improving cognition (13, 14). For example, physical activity increases neurotransmitters such as serotonin and dopamine and helps reduce inflammation linked to depression, supporting its role in intervention (15). Increasing physical activity reduces depression and costs in rural elderly individuals and benefits the mental health of young people (16, 17). Physical exercise and social activities can be used to treat and potentially prevent depression (18). Physical exercise is preferred for treating mild to moderate depression and shows better efficacy than antidepressants do, with minimal side effects (19). Exercise is an effective therapy that positively impacts anxiety disorders and alleviates depression and anxiety symptoms in nonclinical populations (20). A survey of 47 countries with 237,964 respondents revealed that low physical activity increases anxiety disorder risk by 1.32 times (21). Moreover, another meta-analysis suggested that there is insufficient clinical evidence that physical activity significantly reduces anxiety disorders (22). This finding indicates that factors beyond physical activity influence depression and anxiety, with research suggesting that the frequency and intensity of activity affect psychological stress. Inactive individuals tend to struggle with stress management, unlike those who engage in exercise (23). Thus, moderate physical activity effectively reduces stress and enhances overall mental health.

Long-term exercise training can reduce sympathetic nervous system sensitivity, with trained individuals showing lower sympathetic activity and catecholamine levels during exercise than untrained individuals do, indicating better stress management (24). Exercise positively impacts psychological and physiological regulation by decreasing catecholamine levels under chronic stress, improving cardiovascular function, and reducing sympathetic nervous system activation (25). Exercise improves mood and cognition by regulating neurotransmitters such as serotonin and dopamine, alleviating depression and anxiety (26). However, current research has focused mainly on adults and patients with chronic diseases but has not focused on college students, who face significant mental health issues during critical development (27, 28).

### 1.2 The role of diet in depression, anxiety, and stress

Healthier diets enhance exercise's positive effects on mental health, improving self-efficacy and reducing anxiety, whereas malnutrition can diminish exercise enjoyment and affect mental health (29). For example, both the traditional Brazilian diet and olive oil intervention can effectively alleviate anxiety and depression symptoms in severely obese adults (30). In addition, higher dietary quality is negatively correlated with the incidence of severe depression in young people (31). Students who adhere to the Mediterranean diet intake rich fruits, vegetables, and fish, indicating a lower risk of depression. This diet model may have a protective effect on mental health, emphasizing the relationship between eating habits and emotional health (32). The Mediterranean diet is linked to lower anxiety and stress in elderly Australians, but not significantly with depression (33). A negative correlation exists between dietary quality and depression/anxiety symptoms in Chinese adolescents, whereas free sugar intake is positively correlated with these symptoms (34).

Ein et al. (35) demonstrated through a meta-analysis that an 8-week ultralow-calorie diet (≤800 kcal/day), when paired with treatment and exercise, can significantly increase the efficacy of antidepressants (35). Individuals in healthy diet interventions show greater emotional improvement post-aerobic exercise, indicating that good nutrition supports energy and enhances exercise appeal through better psychological states (36). Nutritional improvement reduces depression and anxiety, enhancing the positive mental health impact of exercise (37). Reasonable dietary habits and nutritional support enhance exercise effectiveness and promote neurotransmitter balance. Malnutrition can worsen anxiety and depression, while proper supplementation may alleviate these issues (38). Therefore, good nutrition enhances exercise's psychological benefits and helps establish habits by improving mood and reducing anxiety and depression. Moreover, stress can affect health and quality of life by altering appetite and diet (39, 40). Good nutrition enhances exercise's psychological benefits and helps establish habits by improving mood and reducing anxiety and depression. Moreover, stress can affect health and quality of life by altering appetite and diet (39, 41). For example, the Mediterranean diet, which is high in antioxidants and healthy fats, reduces stress and anxiety, improving mental health (42, 43). Similarly, a diet high in fruits and vegetables is linked to lower psychological stress, suggesting that healthy eating helps relieve stress (44, 45).

### 1.3 Relationship between diet and physical activity

A correlation exists between nutrition and physical activity; university students who exercise tend to follow the Mediterranean diet, improving their nutritional quality (46). However, higher stress and negative emotions, along with lower positive emotions, can reduce physical activity in college students. In contrast, increased activity is associated with lower stress and negative emotions, while also promoting healthier eating habits (core features include low fat, low sugar, low salt, high fruit and vegetable intake, and a preference for natural ingredients) (47). Specifically, physical activity can alleviate stress, reduce negative emotions, and enhance positive emotions, whereas a positive mindset encourages more activity. However, Si and Zhang (48) noted that there is no significant mutual influence between healthy eating and stress or emotional levels. In the elderly population, the combination of a healthy diet (adding dairy, egg, and meat) and moderate physical activity has been shown to significantly lower the risk of mild cognitive impairment, highlighting the potential synergistic effects of diet and exercise in preserving cognitive health (48). In addition, the interaction between diet and exercise cannot be ignored when improving athletic performance. For example, athletes adopting a low-carbohydrate, high-fat diet can improve endurance under specific conditions. However, the adaptation process to this dietary pattern is relatively slow (49). Combining exercise with a balanced diet enhances metabolic benefits and promotes overall health (50). In summary, dietary behavior is linked to physical activity and mediates its effects on mental health issues such as depression and anxiety, making its exploration vital for effective intervention strategies.

Although previous studies have explored the positive effects of physical activity and dietary behavior on mental health, there are still research gaps. First, most studies have focused on the independent effects of a single factor (such as only exercise or diet) on depression, anxiety, and stress, and lack a systematic analysis of the interaction between the two factors. Second, existing research has focused primarily on the general population or chronic disease patients, and there is relatively insufficient research on college students, a special group that is in a critical period of psychological development and has a high incidence of mental health problems. Third, the mediating role of diet between physical activity and mental health has not been systematically studied, which limits a comprehensive understanding of intervention mechanisms. Therefore, this study uses a cross-sectional survey with college students to analyze the relationships among physical activity, diet, and mental health via SEM and mediation analysis, with a focus on dietary behavior as a mediator. This study helps deepen the understanding of the intervention mechanism for college students' mental health and provides a theoretical basis and practical guidance for universities to develop scientifically effective, comprehensive “exercise nutrition” intervention strategies.

## 2 Research participants and methods

### 2.1 Research participants

In total, 1,388 college students from three universities west of China were investigated anonymously via Questionnaire Star. The exclusion criteria for sample screening were as follows: (I) participants were full-time college students; and (II) participants did not have any sports-related disabilities or movement disorders. To guarantee the utmost reliability of the information, prior to completing each questionnaire, an administrator from our team exclusively addressed respondents' inquiries according to the website's guidelines, refraining from offering any leading suggestions. The administrator emphasized that participants should complete the questionnaire independently and clearly stated that they could not access the data or engage in discussions with others. A total of 312 invalid questionnaires were generated due to conflicting viewpoints or other reasons, and 1,076 were ultimately considered valid questionnaires, with an effective rate of 77.5%.

The subjects in this study met the requirements of the Declaration of Helsinki for human experimentation and were approved by the Academic Ethics Committee of Sichuan University of Arts and Sciences (Approval No. 2023SASULL-001). The collected data do not involve sensitive information such as participant names and phone numbers, and we declare that these data are only used for research purposes.

### 2.2 Research methods

#### 2.2.1 Population informatics survey

The demographic information collected in this study included age, sex, major, and grade level.

#### 2.2.2 Adult dietary behavior survey questionnaire

The adult eating behavior questionnaire of Sheng et al. (51), which comprises eight dimensions (I. hunger, II. food reactivity, III. emotional overeating, IV. enjoyment of food, V. satiety, VI. emotional eating deficiency, VII. picky eating, and VIII. delayed eating), has been proven to have good reliability and validity in a survey of the Chinese population (51). All the data were measured using a five-point Likert scale, ranging from “strongly disagree” to “strongly agree.” Cronbach's alpha coefficient was employed to assess the scale's reliability. In this study, the coefficients of the corresponding factors were as follows: hunger 0.828, food reactivity 0.817, emotional overeating 0.924, food enjoyment 0.833, satiety reactivity 0.637, emotional eating 0.948, food pickiness 0.813, delayed eating 0.933, and overall 0.879, indicating the reliability of the scale in this study.

#### 2.2.3 Physical activity survey

The PARS-3 (52) is used to evaluate college students' physical activity in the previous month. This scale consists of three questions, namely, the intensity of physical activity (what is your physical activity or intensity?), duration (how long do you persist in the intensity of the aforementioned physical activity?), and frequency (how many times have you engaged in the aforementioned physical activity?). The corresponding score ranges from 1 to 5 in terms of physical activity levels = intensity score × (duration score – 1) × frequency score, with a score range of 0–100. The scale is widely used in studies of the Chinese population (53, 54). The Cronbach's alpha coefficient was used to evaluate the scale's reliability. In this study, the Cronbach's alpha coefficient was 0.746. This indicates that the scale is reliable.

#### 2.2.4 Depression–anxiety–stress survey

The depression, anxiety, and stress scale (DASS-21) is employed to assess symptoms of depression, anxiety, and stress (55). An early Chinese study verified the reliability of the Chinese version of the tool (56, 57). The students rated 21 statements, with seven measurement questions for each assessed structure (depression, anxiety, and pressure symptoms). All data were measured via a five-point Likert scale, ranging from “strongly disagree” to “strongly agree.” Cronbach's alpha coefficient was employed to assess the scale's reliability. In this study, the Cronbach's alpha coefficients for depression, anxiety, and stress were 0.906, 0.943, and 0.956, respectively, indicating that the scale is reliable.

#### 2.2.5 Data processing

The data analysis was conducted using SPSS 27.0 and Amos 23.0 statistical software. Cronbach's alpha was used to evaluate the reliability of the questionnaire, an independent sample *t*-test was used to analyze the differences in dietary behavior, physical activity, and depression anxiety pressure parameters between male and female college students, the Pearson correlation coefficient was used to measure the relationships between variables, the structural equation measurement model was used to verify the degree of association between variables, and the CMIN/DF, CFI, and RMSEA were used to evaluate the goodness of fit of the model. Differences were considered significant at a *p*-value of ≤ 0.05.

## 3 Results

### 3.1 Basic demographic information

There was no significant difference in age between male and female college students in this survey (18.8 ± 1.0 vs. 18.8 ± 0.7, *p* = 0.733). The proportion of male students is 31.88%, and the proportion of female students is 68.12%. The survey respondents were mainly first-year (44.05%) and second-year university students (44.98%). For specific other information, please refer to Table 1.

**Table 1:** Basic information of the participants.

**Items**	**Sample size**	**%**
**Sex**		
Male	343	31.88
Female	733	68.12
**Major types**		
A	358	33.27
B	331	30.76
C	77	7.16
D	83	7.71
E	227	21.30
**Grade**		
Fresher	474	44.05
Sophomore	484	44.98
Junior	75	6.97
Senior	43	4.00

A: Science and Engineering (Physics, Chemistry, Architecture, Mathematics, and Intelligent Manufacturing); B: Social Sciences (Horse Academy, Foreign Languages, Health and Wellness, and Teacher Education); C: Art (Media, News, Music, and Art); D: Medicine (Clinical, Nursing, and Public Health); E: Physical education.

### 3.2 Differences in diet, physical activity, and depression, anxiety, and stress between male and female college students

Compared to female college students, male college students scored significantly lower in six dimensions: food reactivity, emotional overeating, enjoyability of food, satiety, picky eating, and delayed eating. There were no significant differences in the other two dimensions of the dietary behavior questionnaire. In terms of physical activity, male college students have significantly greater exercise intensity, duration, and frequency than female college students do, and their total physical activity is also significantly greater than that of female college students. There was no significant difference in depression, anxiety, or stress between male and female college students, as shown in Table 2.

**Table 2:** Differences in dietary behavior, physical activity, depression, anxiety, and stress between male and female college students.

**Items**	**Male (*n* = 343)**	**Female (*n* = 733)**	***p-*value**
Hunger	2.9 ± 0.8	2.9 ± 0.8	0.414
Food reactivity	2.9 ± 0.8	3.1 ± 0.9	0.000
Emotional overeating	2.4 ± 1.0	2.7 ± 1.0	0.000
Enjoyability of food	3.9 ± 0.8	4.0 ± 0.8	0.005
Satiety	2.4 ± 0.7	2.7 ± 0.7	0.000
Emotional eating deficiency	2.9 ± 1.1	3.0 ± 1.0	0.103
Picky eating	2.5 ± 0.9	2.7 ± 0.9	0.000
Delayed eating	2.2 ± 0.9	2.4 ± 1.0	0.003
Exercise intensity	3.2 ± 1.3	1.9 ± 1.2	0.000
Exercise duration	4.0 ± 1.2	2.8 ± 1.1	0.000
Exercise frequency	3.4 ± 1.0	2.6 ± 1.0	0.000
Depression	14.9 ± 6.0	15.2 ± 5.5	0.447
Anxiety	16.4 ± 6.9	17.0 ± 6.6	0.212
Stress	14.8 ± 6.3	15.0 ± 6.0	0.576

### 3.3 Relationships among dietary behavior, physical activity, and depression, anxiety, and stress

Through the Pearson correlation analysis, we discovered that the eight dimensions of dietary behavior are positively correlated with depression, anxiety, and stress among college students. Moreover, food enjoyment is inversely related to stress. The level of physical activity is significantly negatively correlated with depression, anxiety, and stress among college students. Among the eight dimensions of dietary behavior, only food reactivity, emotional overeating, satiety, food pickiness, and eating delay are significantly negatively correlated with physical activity levels (the other three dimensions are not correlated with physical activity). See Table 3 for details. Regression analysis was conducted on only the five dimensions of dietary behavior, along with physical activity, depression, anxiety, and stress. Three regression models were established for physical activity, dietary behavior, depression, anxiety, and stress in Models 1–3. In Model 1, the order of weights affecting depression was picky eating, physical activity, emotional overeating, and satiety. In Model 2, the weight order that affects anxiety was picky eating, physical activity, food reactivity, and emotional overeating. In Model 3, the weight order that affects stress was emotional overeating, picky eating, and physical activity. The specific relevant parameters are shown in Table 4.

**Table 3:** Relationships among dietary behavior, physical activity, depression, anxiety, and pressure among college students.

**Items**	**1**	**2**	**3**	**4**	**5**	**6**	**7**	**8**	**9**	**10**	**11**	**12**
1 Hunger	1											
2 Food reactivity	0.571^**^	1										
3 Emotional overeating	0.366^**^	0.456^**^	1									
4 Enjoyability of food	0.236^**^	0.392^**^	0.213^**^	1								
5 Satiety	0.287^**^	0.301^**^	0.265^**^	−0.009	1							
6 Emotional eating deficiency	0.069^*^	0.092^*^	−0.164^**^	−0.043	0.279^**^	1						
7 Picky eating	0.146^**^	0.172^**^	0.151^**^	−0.067^*^	0.288^**^	0.217^**^	1					
8 Delayed eating	0.128^**^	0.136^**^	0.154^**^	−0.076^*^	0.618^**^	0.222^**^	0.287^**^	1				
9 Physical activity	0.011	−0.076^*^	−0.141^**^	−0.004	−0.167^**^	−0.036	−0.099^**^	−0.142^**^	1			
10 Depression	0.131^**^	0.133^**^	0.164^**^	−0.180^**^	0.192^**^	0.146^**^	0.172^**^	0.163^**^	−0.146^**^	1		
11 Anxiety	0.124^**^	0.164^**^	0.163^**^	−0.096^*^	0.147^**^	0.163^**^	0.168^**^	0.127^**^	−0.139^**^	0.772^**^	1	
12 Stress	0.130^**^	0.140^**^	0.192^**^	−0.150^**^	0.149^**^	0.182^**^	0.182^**^	0.150^**^	−0.154^**^	0.772^**^	0.807^**^	1

^*^P < 0.05, ^**^P < 0.01.

**Table 4:** Regression equations of dietary behavior, physical activity, depression, anxiety, and stress.

**Items**	**Unstandardized coefficients**		**Standardized coefficients**	***T***	***p-* value**
	**B**	**Std. Error**	**Beta**		
**Model 1 (vs. Depression)**					
Food reactivity	0.234	0.221	0.036	1.056	0.291
Emotional overeating	0.488	0.185	0.089	2.643	0.008
Satiety	0.602	0.300	0.079	2.008	0.045
Picky eating	0.661	0.196	0.105	3.371	0.001
Delayed eating	0.294	0.218	0.051	1.349	0.178
Physical activity	−0.019	0.006	−0.100	−3.322	0.001
**Model 2 (vs. Anxiety)**					
Food reactivity	0.673	0.263	0.087	2.560	0.011
Emotional overeating	0.514	0.220	0.079	2.342	0.019
Satiety	0.235	0.356	0.026	0.660	0.509
Picky eating	0.834	0.233	0.112	3.578	0.000
Delayed eating	0.272	0.259	0.040	1.051	0.293
Physical activity	−0.023	0.007	−0.100	−3.324	0.001
**Model 3 (vs. Stress)**					
Food reactivity	0.291	0.235	0.042	1.239	0.216
Emotional overeating	0.743	0.196	0.127	3.786	0.000
Satiety	0.038	0.319	0.005	0.118	0.906
Picky eating	0.822	0.208	0.123	3.945	0.000
Delayed eating	0.436	0.232	0.071	1.883	0.060
Physical activity	−0.023	0.006	−0.110	−3.658	0.000

### 3.4 Path analysis of the influence of physical activity on depression, anxiety, and stress in college students

**Figure 1:**
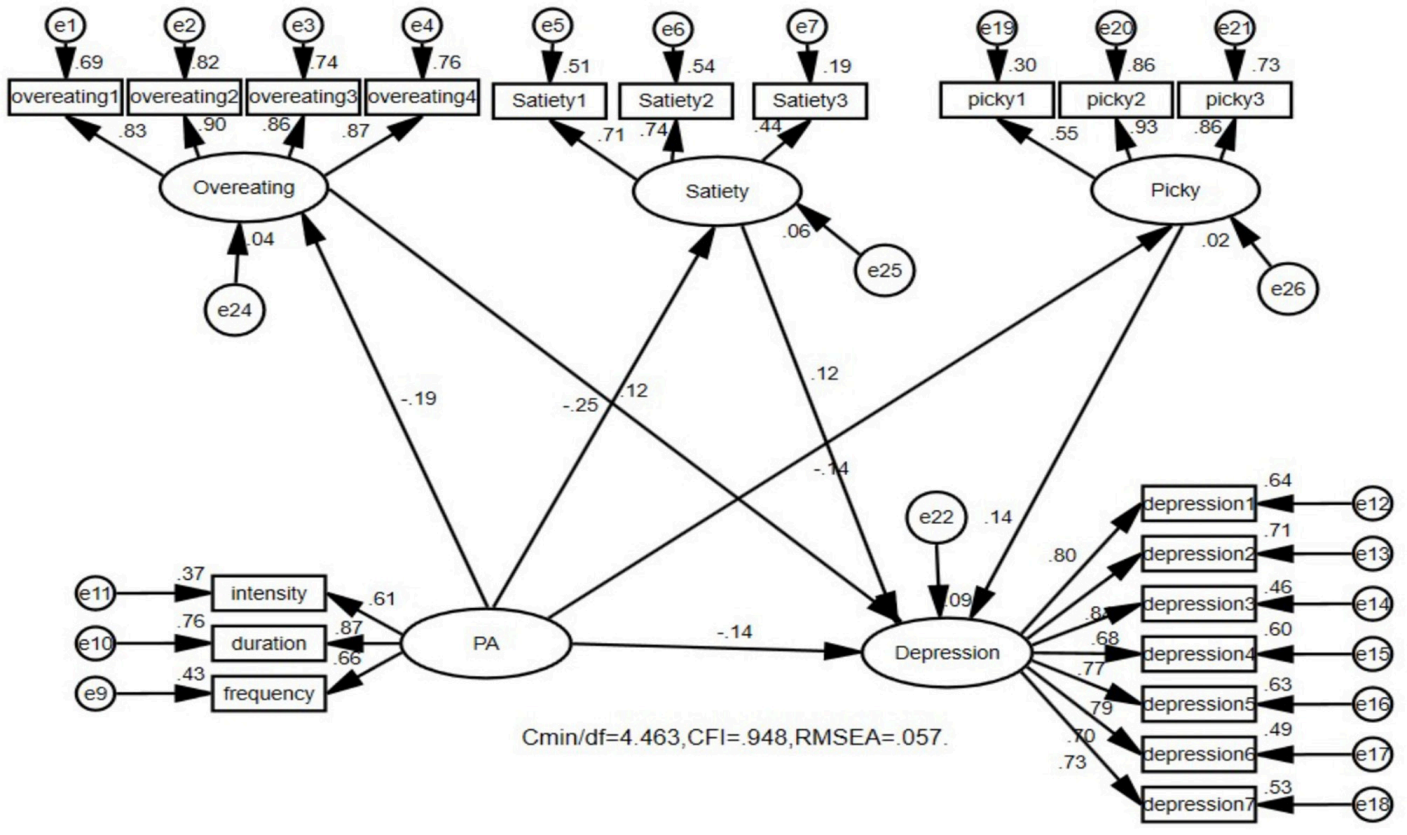
Physical activity not only affects college students' depression through a direct mechanism, but also plays a role through mediating effects from overeating, safety, and picky. The model shows a good fitness coefficient. PA, physical activity.

**Figure 2:**
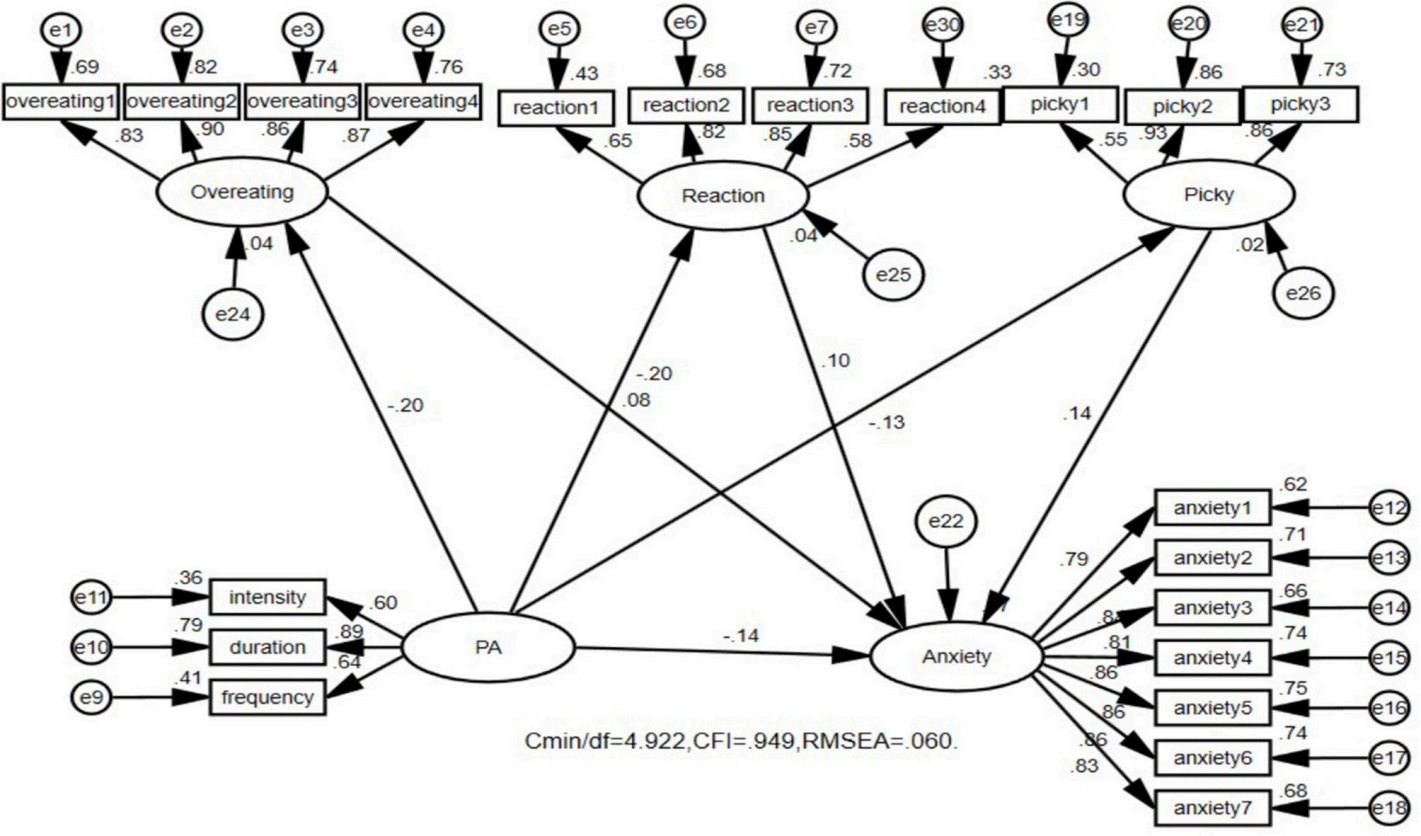
Physical activity not only affects college students' depression through a direct mechanism, but also plays a role through mediating effects from overeating, reaction, and picky. The model shows a good fitness coefficient. PA, physical activity.

**Figure 3:**
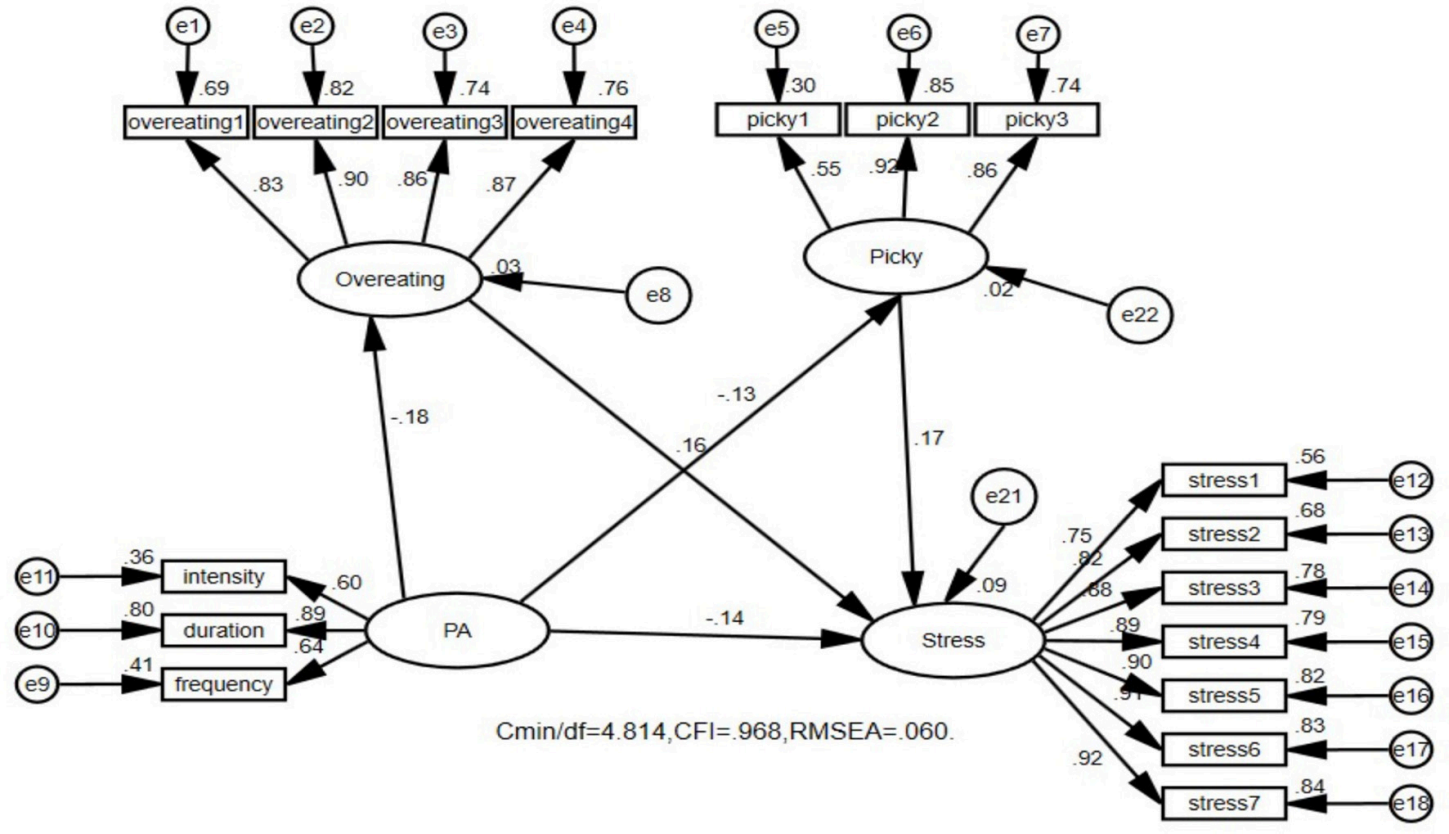
Physical activity not only affects college students' depression through a direct mechanism, but also plays a role through mediating effects from overeating and picky. The model shows a good fitness coefficient. PA, physical activity.

The structural equation model has high flexibility, applicability, and comprehensiveness in path analysis. It can effectively address potential errors and measurement errors and analyze multiple dependent variables and their complex relationships, providing a comprehensive model fitting evaluation. Therefore, the researchers constructed three structural equation models on the basis of the three regression models. Figure 1 shows that physical activity not only directly affects college students' depression but also mediates depression through dietary behavior (Figure 1). The direct effect of physical activity was −0.143, and the 95% confidence interval was (−0.219, −0.064), *p* < 0.01. The mediating effect was −0.053, and the 95% confidence interval was (−0.133, −0.039), *p* < 0.01. The mediating effect was significant; the direct effect accounted for 66.9%, and the mediating effect accounted for 33.1% (see Table 5). Figure 2 shows that physical activity not only directly affects the anxiety level of college students but is also mediated through dietary behavior (Figure 2). The direct effect of physical activity was −0.141, and the 95% confidence interval was (−0.212, −0.069), *p* < 0.01. The mediating effect was −0.071, and the 95% confidence interval was (−0.084, −0.029), *p* < 0.01. The mediating effect was significant; the direct effect accounted for 72.7%, and the mediating effect accounted for 27.3% (see Table 5). Figure 3 shows that physical activity not only directly affects the stress level of college students but is also mediated through dietary behavior (Figure 3). The direct effect of physical activity was −0.136, and the 95% confidence interval was (−0.204, −0.068), *p* < 0.01. The mediating effect was −0.049, and the 95% confidence interval was (−0.078, −0.027), *p* < 0.01. The mediating effect was significant; the direct effect accounted for 73.5%, and the mediating effect accounted for 26.5% (see Table 5).

**Table 5:** Path analysis of physical activity and dietary behavior influencing depression, anxiety, and stress.

**Effect**	**Path**	**Beta**	***p-*value**	**95%CI**	
				**Low**	**Upper**
Direct effect	PA- Depression	−0.143	0.002	−0.219	−0.064
Indirect effect	PA- DB1-Depression	−0.071	0.001	−0.113	−0.039
Total effect	PA- Depression	−0.214	0.003	−0.275	−0.137
Direct effect	PA- Anxiety	−0.141	0.000	−0.212	−0.069
Indirect effect	PA- DB2- Anxiety	−0.053	0.000	−0.084	−0.029
Total effect	PA- Anxiety	−0.194	0.000	−0.262	−0.124
Direct effect	PA- Stress	−0.136	0.000	−0.204	−0.068
Indirect effect	PA- DB3- Stress	−0.049	0.000	−0.078	−0.027
Total effect	PA- Stress	−0.185	0.000	−0.252	−0.118

DB1 is composed of three intermediaries, namely, overeating, satiety, and picky; DB2 is composed of three intermediaries, namely, overeating, reaction, and pickiness; DB3 is composed of two intermediaries, namely, overeating and picky eating.

PA, physical activity.

## 4 Discussion

### 4.1 Main findings of this study

This study revealed significant differences in dietary behavior (food reactivity, emotional overeating, enjoyability of food, satiety, picky eating, and delayed eating) and physical activity between male and female college students. However, no significant differences were observed in depression, anxiety, or stress scores. Physical activity directly influences depression and anxiety levels and mediates these effects through dietary behavior, reducing depression, anxiety, and stress.

### 4.2 Gender differences in physical activity and dietary behavior

Some men prefer high-intensity exercise, such as strength training, whereas some women prefer low-intensity activities, such as walking and yoga (58, 59). Men have a higher physical activity level than women do and are more likely to meet WHO recommendations (60, 61). Male college students scored higher than female college students did in terms of physical activity intensity, duration, frequency, and level, which aligns with past research (59, 60). Differences among college students stem from biological and sociocultural factors; men favor high-intensity exercise, whereas women prefer low-intensity exercise (58, 59). Traditional beliefs link “masculinity” to physical ability, prompting men to engage in intense exercise, whereas women prefer low-calorie workouts due to beauty standards (58, 60). Men prefer high-energy foods and have limited dietary options (59, 60, 62). Women are more prone to emotional eating, whereas men eat mainly due to hunger, indicating different emotional and physiological mechanisms (59, 63).

Compared to male college students, female college students presented greater food reactivity, emotional overeating, enjoyment of food, satiety, and picky eating, which aligns with the findings of Berge et al. (63) and Lombardo et al. (59). These dietary gender differences may stem from women's heightened sensitivity to food odors and tastes due to estrogen fluctuations, which impact satiety signals (58, 59). Women are more sensitive to leptin, but ghrelin levels fluctuate with emotions, causing emotional eating (59, 63). Women often cope with negative emotions by eating, whereas men prefer exercise or social activities, highlighting gender differences in emotional regulation (58, 59, 63). Future interventions should include mindfulness eating training for women, address “health food” stereotypes with education, and reduce the risk of imbalanced diets resulting from food neophobia.

### 4.3 Positive effects of physical activity on depression, anxiety, and stress among college students

Recent reviews have shown that physical activity, including aerobic exercise, strength training, or yoga, effectively alleviates depressive symptoms, comparable to medications or therapy (64, 65). In contrast, dietary interventions can reduce depression in non-clinical and female groups but are ineffective for anxiety, with unclear active ingredients and mechanisms (66). The intake of ultra-processed foods (UPFs) is linked to increased depression risk, indicating a need to reduce ultra-processed foods (UPFs) while increasing exercise (67). Moderate-intensity continuous training and high-intensity interval training (HIIT) (HIIT) have comparable effects, but HIIT is more time-efficient; yoga efficacy depends on frequency and cultural adaptation (68). Overall, physical activity is recommended for depression prevention and treatment because of its low cost and risk. However, high-quality randomized controlled trials are needed to determine the optimal dosage and personalization.

A negative correlation (*r* = −0.139, *p* < 0.01) between physical activity and anxiety was found in college students, which is consistent with the findings of a recent study in which exercise's antianxiety efficacy was comparable to that of pharmacological treatments or therapy, with no addiction risk or benefits for cardiovascular issues (69). The dose–response relationship suggests that moderate-to-high-intensity aerobic exercise (≥60% VO_2_ peak, ≥150 min per week) or high-intensity interval training (HIIT) can significantly reduce state–trait anxiety scores within 2–4 weeks, whereas low-intensity activities (such as yoga) require more extended intervention periods to achieve equivalent results (70). Physical activity significantly affects anxiety disorders more than depression does, indicating that anxiety symptoms may be more sensitive to exercise-induced neurochemical changes. The molecular mechanisms by which exercise mitigates anxiety can be encapsulated by the three interdependent pillars of “monoamine inhibition plasticity” (71–73). The phased release of the dopamine reward system elevates mood and establishes a positive feedback loop that reinforces exercise behavior, further improving adherence to the intervention (74).

This investigation did not reveal a notable association between physical activity and subjective stress levels among college students. Nonetheless, prior research has indicated that exercise may modulate the stress response via a biphasic process characterized by “acute activation and chronic adaptation.” Acute high-intensity exercise instantly increases adrenaline and noradrenaline levels, enhancing energy mobilization and alertness (75). However, endurance training reduces peak catecholamine secretion from rest and stress, increasing heart rate variability, indicating synergistic adaptation with lower sympathetic sensitivity and higher parasympathetic tone (24, 76). This adaptive modification is crucial for chronically stressed populations, as regular physical activity can reduce cardiovascular risk by 10%−20%, similar to beta-blockers (25). Therefore, the lack of correlation in cross-sectional studies may be due to low sample pressure or imprecise exercise volume measurement, but not biological pathway failure. Alkhatatbeh et al. (77) confirmed that young adults exhibiting suboptimal sleep quality tend to experience heightened levels of anxiety and depression in comparison to their well-rested counterparts, which is often accompanied by more pronounced musculoskeletal discomfort. Enhancing sleep quality may be a potential intervention to mitigate these adverse symptoms (77). Furthermore, a dose–response relationship exists between deteriorating sleep quality and the intensification of depressive symptoms, wherein poorer sleep correlates with increased severity of depression (78). Sleep disorders not only constitute a significant risk factor for depression but also may intensify preexisting symptoms (79).

Prolonged engagement with electronic devices is recognized as a critical element influencing sleep quality, particularly among adolescents. Empirical studies have demonstrated a robust correlation between the utilization of electronic devices and the prevalence of sleep disorders (80, 81). Excessive use of smartphones and computers is positively associated with depressive symptoms, revealing a notable increase in the incidence of depression and anxiety among adolescents who engage with these devices for durations exceeding 2 h (82, 83). Moreover, interaction with electronic devices may adversely affect adolescents' social skills and psychological resilience, further aggravating emotional challenges.

### 4.4 Dietary behavior plays a mediating role in the relationships among physical activity, depression, anxiety, and stress among college students

Numerous studies have demonstrated that engaging in physical exercise serves as a potent strategy for mitigating the risks of anxiety and depression (84–86). Enhanced physical activity significantly reduces anxiety and depression, with prior research showing a strong link between higher activity levels and lower symptoms. Moreover, sleep quality also negatively correlates with both conditions (87). Increased physical activity is linked to better wellbeing and sleep quality, indicating that sleep mediates the relationship between activity and anxiety (88). However, diverse viewpoints persist in this domain; for example, (22) established that there is a lack of sufficient evidence to substantiate the claim that physical activity can effectively avert the onset of anxiety disorders (22).

In this research, we discovered that engaging in physical activity can influence the levels of depression and anxiety among college students, while dietary measures can effectively modulate these conditions. This finding validates previous scholars' views (31, 36) and introduces a novel perspective that dietary habits significantly mediate this relationship. Physical activity regulates energy expenditure, appetite, and ghrelin, reducing emotional eating and mitigating the effects of high sugar on serotonin and dopamine (59). Exercise enhances leptin sensitivity and reduces the risk of depression linked to eating disorders (89). Regular physical activity enhances self-efficacy and reduces stress-related eating behaviors. This change effectively interrupts the vicious cycle characterized by pressure → emotional eating → guilt → anxiety (59, 63). Adverse emotions can lead to unhealthy eating and increased distress, whereas exercise encourages better food choices and emotional control (90). A prior investigation revealed a reciprocal relationship between the quality of sleep and states of depression and anxiety (88). Future studies should assess the relationships among physical activity, diet, sleep, and mental health. Supplementation may reduce inflammation linked to high-fat diets. Changes in the gut microbiota can affect cognition and lead to anxiety or depression (91). These investigations provide new ways to manage depression and anxiety.

Physical activity is crucial for an active lifestyle and significantly reduces stress, especially in college students, who experience lower stress levels with increased exercise (92). This study confirmed that college students' physical activity not only directly affects stress but also exerts its effect through the mediating role of overeating and pickiness. An additional study revealed that physical exercise is a strong stress management strategy that helps students face challenges and maintain mental wellbeing (93). Physical activity alleviates stress, but the role of dietary habits is often overlooked; nutrition affects both diet quality and mental wellbeing, with studies showing a strong link between diet and mental health, especially under pressure (94). For example, emotional eating involves the use of food to cope with stress, leading to harmful habits and increased psychological distress (95).

Compared to their sedentary peers, students who exercised regularly presented better nutrition and lower stress (92). Dietary improvements can significantly reduce stress, especially in physically active individuals (96). Dietary habits partially mediate the link between physical activity and stress, with improved nutrition, especially more fruits and vegetables, increasing mental wellbeing and reducing anxiety and depression (97). In addition, a nutritious diet positively correlates with increased physical activity, suggesting that better dietary habits can reduce stress levels (96). Gender significantly influences physical activity and stress management, with women seeking social support and men preferring exercise (96). Women seek social support under stress, whereas men relieve tension through physical activity (98). Therefore, the formulation of tailored exercise regimens that consider individual capabilities and preferences can significantly enhance stress management.

### 4.5 Innovations and limitations of this study

The originality of this study is reflected in the following three points: (I) the initial identification of an absence of substantial gender disparities in the levels of depression, anxiety, and stress among college students. Concurrently, a pronounced gender distinction in dietary habits and physical activity emerges, thereby contesting the conventional reliance on gender stereotypes in mental health interventions; (II) the innovative validation of the impact of physical activity on mental health through direct effects (depression/anxiety) and indirect mediation (dietary behavior), breaking through the previous research framework of single behavioral effects; and (III) the first empirical evidence shows that emotional overeating and food preferences can explain 75% of the variation in stress levels, overturning the notion that physical activity dominates stress regulation and establishing the core position of diet in stress management.

The current investigation serves as a trailblazer in revealing gender-invariant levels of depression, anxiety, and stress while simultaneously revealing gender-specific patterns in dietary habits and physical activity. It establishes direct and indirect pathways (mediated through dietary behaviors) through which physical activity influences mental health. However, the cross-sectional design somewhat constrains the efficacy of causal inference, particularly under the assumption that diet is a mediator between physical activity and psychological and emotional symptoms; it fails to eliminate the potential influences of reverse causality and unmeasured confounding factors on path coefficients. Future investigations should embrace longitudinal or experimental frameworks to elucidate the temporal and causal relationships, augmented by sensitivity analyses of the reverse path model to assess the robustness of the mediation hypothesis. Self-reported data are prone to various biases, including response, comprehension, and recall biases—limitations that could be alleviated by incorporating ecological momentary assessments, wearable technology, and digital food diaries for the continuous, objective tracking of physical activity and nutritional intake. The limited sociocultural diversity of the current sample restricts the generalizability of the findings; therefore, multisite, cross-cultural, and lifespan studies are warranted to evaluate the boundary conditions of the present results. Finally, the simplistic operationalization of diet inhibits the identification of specific foods, nutrients, or dietary patterns that contribute to stress; future research should utilize comprehensive dietary records, biomarkers (such as inflammatory cytokines and gut microbiome profiles), and nutrient databases to develop multifaceted “food–nutrient–metabolome–mood” frameworks, thereby translating our findings into precise, tailored nutritional strategies for the enhancement of mental health.

## 5 Conclusion

This study systematically evaluated differences in dietary habits, physical exercise, and levels of depression, anxiety, and stress between male and female college students, while exploring the potential impact of dietary habits on the relationship between physical activity and mental health (including depression, anxiety, and stress). The results reveal the complex interaction between students' lifestyles and mental health indicators, providing valuable cross-sectional insights into the mechanisms by which physical exercise and nutrition promote mental health.

## Data Availability

The raw data supporting the conclusions of this article will be made available by the authors, without undue reservation.
